# Nucleolar Phosphoprotein NPM1 Interacts With Porcine Circovirus Type 3 Cap Protein and Facilitates Viral Replication

**DOI:** 10.3389/fmicb.2021.679341

**Published:** 2021-05-25

**Authors:** Jiangwei Song, Lei Hou, Dan Wang, Li Wei, Shanshan Zhu, Jing Wang, Rong Quan, Haijun Jiang, Ruihan Shi, Jue Liu

**Affiliations:** ^1^Beijing Key Laboratory for Prevention and Control of Infectious Diseases in Livestock and Poultry, Institute of Animal Husbandry and Veterinary Medicine, Beijing Academy of Agriculture and Forestry Sciences, Beijing, China; ^2^College of Veterinary Medicine, Yangzhou University, Yangzhou, China; ^3^Jiangsu Co-innovation Center for Prevention and Control of Important Animal Infectious Diseases and Zoonoses, Yangzhou University, Yangzhou, China

**Keywords:** capsid protein, interaction, NPM1, porcine circovirus type 3, replication

## Abstract

Porcine circovirus type 3 (PCV3) is a recently discovered virus with potentially significant implications on the global swine industry. PCV3 replication involves the entry of the viral capsid (Cap) protein with nucleolar localization signals into the nucleus. Using liquid chromatography-mass spectrometry analysis, nucleolar phosphoprotein NPM1 was identified as one of the cellular proteins bound to PCV3 Cap. Co-immunoprecipitation demonstrated that PCV3 Cap interacts directly with NPM1, where the region binding with NPM1 is mapped to amino acid residues 1–38 of Cap. Upon co-transfection, the expression of Cap protein promoted the redistribution of NPM1, which translocated from the nucleus to the cytoplasm and colocalized with Cap in cultured PK15 cells. NPM1 expression was upregulated and translocated from the nucleus to the cytoplasm in PCV3-infected cells, upon siRNA-mediated depletion, or upon treatment with NPM1 inhibitor in PK15 cells with impaired PCV3 replication, as evidenced by decreased levels of viral DNA synthesis and protein expression. By contrast, the replication of PCV3 was enhanced in stably NPM1-expressing cells via a lentivirus-delivered system. Taken together, these findings indicate that NPM1 interacts with PCV3 Cap and plays a crucial role in PCV3 replication.

## Introduction

Porcine circovirus (PCV), a member of the genus *Circovirus* of the family *Circoviridae*, is a non-enveloped, single-stranded, and closed-circular DNA virus with genomes of approximately 1.7–2.0 kb in length (Breitbart et al., 2017). To date, four species of PCVs have been identified: porcine circovirus type 1–4 (PCV1 to PCV4) (Oh and Chae, 2020; Zhang et al., 2020). Emerging PCV3 is related to porcine dermatitis and nephropathy syndrome (PDNS)-like clinical disease and multisystemic diseases in piglets and sows (Phan et al., 2016; Palinski et al., 2017; Jiang et al., 2019). The PCV3 genome contains three major open reading frames (ORFs), namely ORF1, ORF2, and ORF3, which encode replicase (Rep), structural capsid protein (Cap), and an unknown function ORF3 protein, respectively (Li et al., 2018). Without encoding DNA polymerase in circoviruses, the replication machinery of the host cell is needed for *de novo* DNA synthesis (Heath et al., 2006). All PCVs possess a nuclear localization signal (NLS) at the N-terminus of the Cap protein (Liu et al., 2001; Shuai et al., 2008; Mou et al., 2019). Although the amino acids of the PCV3 Cap protein are significantly different from those of PCV1 and PCV2, their motifs within the NLSs share some similarities (Liu et al., 2001; Shuai et al., 2008; Mou et al., 2019).

NLSs are considered important components of DNA viruses, RNA viruses, and retrovirus-encoding proteins (Matthews, 2001; Wurm et al., 2001; Hiscox, 2002; Salvetti and Greco, 2014; Passos-Castilho et al., 2018). These NLS-containing viral proteins play a prominent role in virus transcription and translation, as well as cell cycle and division (Matthews, 2001; Wurm et al., 2001; Hiscox, 2002; Salvetti and Greco, 2014; Passos-Castilho et al., 2018). Specific viral proteins encoding nucleolar localization signals target the nucleolus during viral replication, effectively inducing changes in the nucleolar morphology and organization of cells (Matthews, 2001; Wurm et al., 2001; Hiscox, 2002; Salvetti and Greco, 2014; Passos-Castilho et al., 2018). The NLS plays an important role in modulating the life cycle of PCV2 by interacting with nucleolar phosphoprotein nucleophosmin-1 (NPM1), which helps Cap shuttle from the nucleolus to the cytoplasm (Zhou et al., 2020). A previous study showed that expression of NPM1 in the lymph nodes of PCV2-infected piglets was slightly increased (Ramirez-Boo et al., 2011). NPM1, also known as NO38, B23, or numatrin, participates extensively in diverse cellular processes, including RNA transcription, ribosome assembly and biogenesis, DNA replication and repair, translation, regulation of cell growth, and nucleocytoplasmic transport (Colombo et al., 2011; Lindstrom, 2011). By acting as a nuclear shuttle phosphoprotein, NPM1 resides predominantly in the nucleolus and is composed of an N-terminal oligomerization domain, a central histone-binding domain, and a C-terminal RNA-binding domain (Yun et al., 2003; Okuwaki, 2008). NPM1 has also been demonstrated to contribute to various stages of viral infection by binding to a multiple of viral proteins, including human immunodeficiency virus type 1 (HIV-1) Rev (Fankhauser et al., 1991), adenoviral core (Samad et al., 2007), human T-cell leukemia virus type 1 (HTLV-1) Rex (Adachi et al., 1993), herpes simplex virus type 1 (HSV-1) UL24 (Lymberopoulos et al., 2011), Japanese encephalitis virus (JEV) core (Tsuda et al., 2006), and Epstein-Barr virus nuclear antigen 2 (Liu et al., 2012). Zhou et al. (2021) recently reported that NPM1 interacts with the PCV3 Cap and contributes to its nucleolar localization. However, whether NPM1 contributes to PCV3 replication remains unclear.

In the present study, immunoprecipitation coupled with liquid chromatography-mass spectrometry (LC-MS/MS) was used to identify the host cellular proteins interacting with PCV3 Cap. In the protein interacting network, NPM1 was found to bind to PCV3 Cap at its N-terminus by co-immunoprecipitation (Co-IP). Furthermore, infection with PCV3 was found to upregulate the expression of NPM1 in cultured cells, and the expression of Cap protein induced the redistribution of NPM1 from the nucleus to the cytoplasm. Silencing the expression of NPM1 resulted in a significant reduction of PCV3 replication, whereas NPM1 overexpression promoted PCV3 replication. These results suggest that NPM1 plays a pivotal role in the replication of PCV3.

## Materials and Methods

### Cells, Viruses, Reagents, and Antibodies

PK15 cells were cultivated in Dulbecco’s modified Eagle medium (DMEM) (Invitrogen, CA, United States) supplemented with 10% fetal bovine serum (FBS) (Invitrogen), and maintained in an incubator at 37°C with 5% CO_2_. The PCV3 strain LY (Jiang et al., 2019) was used. NSC348884 was purchased from MedChemExpress (Monmouth Junction, NJ, United States). FLAG mAb (F1804), FLAG pAb (F7425), HA mAb (H3663), and β-actin mAb (A1978) were purchased from Sigma-Aldrich (St. Louis, MO, United States). NPM1 mAb (ab10530), green fluorescent protein (GFP) mAb (ab127417), and histone H3 pAb (ab183902) were purchased from Abcam (Cambridge, MA, United States). GFP pAb (50430-2-AP) and NPM1 pAb (10306-1-AP) were purchased from Proteintech (Rosemont, IL, United States). HA pAb (3724) and nucleolin pAb (14574) was purchased from Cell Signaling Technology (Danvers, MA, United States). Cap mAb was prepared in our laboratory. Goat anti-rabbit IgG (Horseradish peroxidase- conjugated, HRP) (ab205718) and goat anti-mouse IgG (HRP) (ab205719) were purchased from Abcam. Goat anti-mouse secondary antibody (Alexa-fluor-488), goat anti-mouse secondary antibody (Alexa-fluor-568), and goat anti-rabbit secondary antibody (Alexa-fluor-568) were purchased from Molecular Probes (Invitrogen).

### Plasmid Construction

Plasmids expressing the *Cap* gene were constructed by cloning from the PCV3 infectious clone into pCMV-HA (#631604; Clontech), pEGFP-C1 (#U55763; Clontech), and p3 × FLAG-CMV-10 (#E4401; Sigma-Aldrich) vectors. The NPM1, nucleolin, and fibrillarin genes from PK15 cells were amplified and recombined into the lentivirus vector pWPXL (#12257; Addgene) to express the fusion proteins NPM1-GFP, nucleolin-GFP, and fibrillarin-GFP. Lipofectamine^TM^ LTX Reagent (#A12621; Thermo Fisher Scientific) was used for plasmid transfection. The primers are displayed in Table 1.

**TABLE 1:** Primers used in this study.

**Primers^a^**	**Sequence (5′–3′)^b^**	**Restriction site**
GFP-Cap-F	TCGAGCTCAAGCTTCGAATTCTATGAGACACAGAGCTATATTCAGA	EcoRI
GFP-Cap-R	TTATCTAGATCCGGTGGATCCTTAGAGAACGGACTTGTAACGAAT	BamHI
GFP-Cap(1–38)-F	TCGAGCTCAAGCTTCGAATTCTATGAGACACAGAGCTATATTCAGA	EcoRI
GFP-Cap(1–38)-R	TTATCTAGATCCGGTGGATCCTTAGTATGTGCCAGCTGTGGGCCTCCT	BamHI
GFP-Cap(39–106)-F	TCGAGCTCAAGCTTCGAATTCTTACACAAAGAAATACTCCACCATG	EcoRI
GFP-Cap(39–106)-R	TTATCTAGATCCGGTGGATCCTTAGTGCCCGAACATAGTTTTTGTT	BamHI
GFP-Cap(107–174)-F	TCGAGCTCAAGCTTCGAATTCTACAGCCATAGATCTAGACGGCGC	EcoRI
GFP-Cap(107–174)-R	TTATCTAGATCCGGTGGATCCTTAGTTGAGCCATGGGGTGGGTCTG	BamHI
GFP-Cap(175–214)-F	TCGAGCTCAAGCTTCGAATTCTACATATGACCCCACCGTTCAATGG	EcoRI
GFP-Cap(175–214)-R	TTATCTAGATCCGGTGGATCCTTAGAGAACGGACTTGTAACGAAT	BamHI
HA-Cap-F	TGGCCATGGAGGCCCGAATTCGGATGAGACACAGAGCTATATTCAGA	EcoRI
HA-Cap-R	GATCCCCGCGGCCGCGGTACCTTAGAGAACGGACTTGTAACGAATCCA	KpnI
Flag-Cap-F	CAAGCTTGCGGCCGCGAATTCAATGAGACACAGAGCTATATTCAGA	EcoRI
Flag-Cap-R	CCTCTAGAGTCGACTGGTACCTTAGAGAACGGACTTGTAACGAAT	KpnI
HA-NPM1-F	TGGCCATGGAGGCCCGAATTCGGATGGAAGATTCGATGGATATG	EcoRI
HA-NPM1-R	GATCCCCGCGGCCGCGGTACCTTAAAGAGACTTCCTCCACTGC	KpnI
GFP-NPM1-F	GAGGTTTAAACTACGGGATCCAATGGAAGATTCGATGGATATGGAC	BamHI
GFP-NPM1-R	ACCGGTAGCGCTAGGACGCGTAAAAGAGACTTCCTCCACTGCCAGAG	MluI
FP-Nucleolin-F	GAGGTTTAAACTACGGGATCCAATGGTAAAGCTCGCAAAGGCCG	BamHI
GFP-Nucleolin-R	ACCGGTAGCGCTAGGACGCGTAATTCAAACTTCGTCTTCTTTCCTTG	MluI
GFP-Fibrillarin-F	GAGGTTTAAACTACGGGATCCAATGAAACCAGGTTTCAGCCCCC	BamHI
GFP-Fibrillarin-R	ACCGGTAGCGCTAGGACGCGTAAGTTCTTCACCTTGGGGGGTGGCCT	MluI
Cap-F	GTGCCAGGGCTTGTTATTCT
Cap-R	CTATTCATTAGGAGGCCCACAG
NPM1-F	CTCAAAACCGTCAACACCAA
NPM1-R	CCGGAAACAATTCTTCACATAA
GAPDH-F	ATCCCGCCAACATCAAATG
GAPDH-R	TACTTCTCATGGTTCACGC

*^*a*^F denotes forward PCR primer; R denotes reverse PCR primer. ^*b*^Restriction sites are underlined.*

### Silver Staining and Mass Spectrometric Identification Proteins

PK15 cells were lysed with NP 40 buffer (10 mM Tris pH 7.5, 150 mM NaCl, 0.5% NP-40, 0.5 mM EDTA) and incubated with anti-GFP mAb conjugated beads (#gtma10, Chromotek) after 36 h of transfection with plasmids expressing GFP-Cap or GFP. The immunoprecipitated proteins were washed with washing buffers (10 mM Tris pH 7.5, 150 mM NaCl, 0.05% NP-40, 0.5 mM EDTA) three times and separated by sodium dodecyl sulfate-polyacrylamide gel electrophoresis (SDS-PAGE) and then visualized using a silver stain kit (#24612; Thermo Fisher Scientific) in accordance with the manufacturer’s instructions. The differential bands in the lane for transfected GFP-Cap and the gel parallel to the lane for transfected GFP were manually excised and subjected to LC-MS/MS analysis in Aptbiotech (Shanghai, China) as previously described (Zhang et al., 2009).

### Co-IP

The whole cell lysates were extracted from co-transfected HEK293FT cells using NP-40 buffer (10 mM Tris pH 7.5, 150 mM NaCl, 0.5% NP-40, 0.5 mM EDTA) for 30 min at 4°C, then centrifuged at 13,000 rpm for 20 min. The clarified samples were incubated with Pierce Anti-HA Magnetic Beads (#88836; Thermo Fisher Scientific) overnight at 4°C with rotation. The immunoprecipitation pellets were collected by DynaMag-2^TM^ Magnet (#12321D; Thermo Fisher) and washed three times, followed by western blot analysis.

### PCV3 Infections

Monolayers of PK15 cells were infected with PCV3 strain LY followed by additional treatment with 300 mM D-glucosamine at 18–24 h after infection as described previously (Jiang et al., 2019).

### Western Blotting

The cells at the indicated times either post-infection or post-transfection were lysed using NP-40 buffer for 30 min at 4°C. The whole cell lysates were centrifuged at 13,000 rpm for 20 min. The concentration of proteins was quantified using the bicinchoninic acid (BCA) protein assay kit (#23225; Thermo Fisher Scientific). Protein samples were separated by SDS-PAGE, then transferred to polyvinylidenedifluoride membranes (Millipore, United States), followed by incubation with primary antibody for 4 h. After washing, the membranes were incubated with secondary antibody for 1 h. Protein bands were detected using ECL detection reagents (34096; Thermo Fisher Scientific).

### Quantitative Real-Time RT-PCR (RT-qPCR)

At the indicted times following PCV3 infection, total RNA was isolated by TRIzol reagent (Invitrogen). A thousand nanograms of RNA sample was used for first stranded cDNA synthesis by using SuperMix for qPCR (Vazyme, Nanjing, China). Real-time PCR was performed on an iQ5 real time PCR system (Bio-Rad) using a One-Step RT-PCR Kit with SYBR Green (Bio-Rad), according to the manufacturer’s instructions. The primers used are listed in Table 1. The glyceraldehyde-3-phosphate dehydrogenase (GAPDH) gene was used as an internal control. The relative fold changes of mRNA were quantified by threshold cycle (CT) (2^–ΔΔCT^) method (Schmittgen and Livak, 2008).

### Quantification of PCV3 DNA by Real-Time PCR

Total DNA was isolated from PCV3-infected PK15 cells using a DNeasy Blood and Tissue Mini kit (#69504; Qiagen) in accordance with the manufacturer’s instructions. A region from PCV3 *Cap* gene was amplified using a real-time PCR method described in a recent study (Jiang et al., 2019).

### Immunofluorescence Assays (IFA)

At the indicated times either post-infection or post-transfection, the cells were washed with PBS and fixed with 3.7% paraformaldehyde for 10 min, then washed three times with PBS and permeabilized with 0.1% Triton-X 100 in 2% bovine serum albumin (BSA) for 10 min, and followed by blocking with 2% BSA in PBS for 30 min. Primary antibodies were incubated for 1 h, and washed with PBS for three times. Next, the cells were incubated with the appropriate secondary antibodies for 1 h. Nuclei were stained with 4′,6-diamidino-2-phenylindole (DAPI) to obtain images using a Nikon Al confocal microscope.

### Nuclear and Cytoplasmic Protein Extraction

PK15 cells seeded in 6-well plates were transfected with plasmids expressing GFP-Cap and GFP. At 48 h of transfection, the cells were washed with PBS, harvested with trypsin digestion, and then centrifuged at 2,500 rpm for 2 min. NE-PER Nuclear and Cytoplasmic Extraction Reagents (#78833; Thermo Fisher Scientific) was used for nuclear and cytoplasmic protein extraction, respectively. Histone-H3 and β-actin were used as markers for nuclear and cytoplasm fractions quality by Western blot analysis.

### Lentivirus Transduction

Three lentivirus packaging plasmids pWPXL, pMD2.G, and psPAX2 were purchased from Addgene (Watertown, MA, United States). The three plasmids were co-transfected into HEK293FT cells using Lipofectamine^TM^ LTX Reagent (#A12621; Thermo Fisher Scientific). The supernatants were harvested at 36 h post-transfection, filtered, concentrated, and titrated. PK15 cells seeded in 24-well plates were transduced with rescued lentiviruses in the presence of polybrene.

### RNA Interference Assay

Small interfering RNAs (siRNAs) targeting NPM1 were designed to three different coding regions, including siNPM1-304 (sense, 5′-CCACUUUAAGGUGGAUAAUTT-3′; antisense, 5′-AUUAU CCACCUUAAAGUGGTT-3′), siNPM1-407 (sense, 5′-GCAAU GAAUUAUGAAGGCATT-3′; antisense, 5′-UGCCUUCAUAAU UCAUUGCTT-3′), siNPM1-577 (sense, 5′-GGAAGAUGCAGA GUCAGAATT-3′; antisense, 5′- UUCUGACUCUGCAUCUUC CTT-3′), and negative control siRNAs (siNC) (sense, 5′-UU CUCCGAACGUGUCACGUTT-3′; antisense, 5′-ACGUGACA CGUUCGGAGAATT-3′). The synthesis of siRNAs from GenePharma (Suzhou, China). Lipofectamine RNAiMAX (#13778150; Thermo Fisher Scientific) was used for siRNAs transfection.

### Statistical Analysis

All statistical analyses were performed using GraphPad Prism version 5.0 (La Jolla, CA, United States). Data are expressed as the mean ± standard deviation (SD). Statistical significance was determined using two-tailed unpaired Student’s *t*-test and two-way analysis of variance (ANOVA).

## Results

### Gene Ontology (GO) and Kyoto Encyclopedia of Genes and Genomes (KEGG) Pathway Analysis of Cellular Proteins Interacting With PCV3 Cap

**FIGURE 1:**
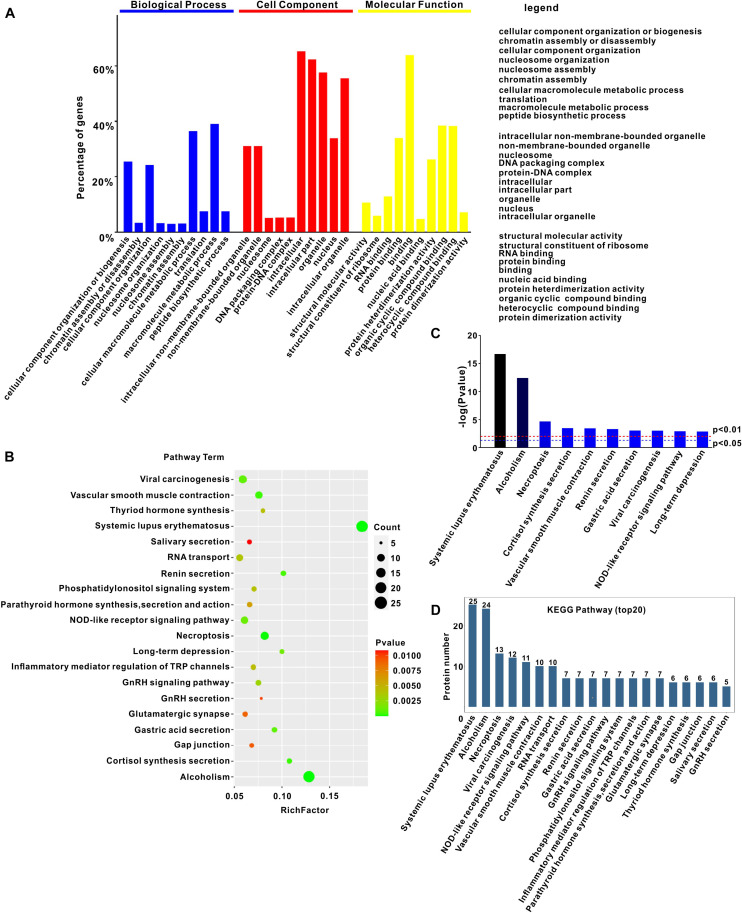
Gene ontology and KEGG pathway enrichment analysis of interactions. **(A)** Annotation of proteins interacting with PCV3 Cap using Gene Ontology. **(B)** KEGG pathway analysis of cellular proteins interacting with PCV3 Cap. **(C)** Enrichment of differential pathway terms in KEGG pathways. Cut-off *P*-value of 0.01 was used to reduce false discovery rate. **(D)** Top 20 KEGG pathways.

The LC-MS/MS approach was used to identify the host proteins interacting with PCV3 Cap. To this end, a GFP-fused Cap was transfected into PK15 cells and a GFP empty vector was used as a control. At 24 h after transfection, the GFP-trap beads were applied to pull down the interacting host proteins, followed by silver staining. The distinct bands in the GFP-Cap transfected group and the control group in the parallel area were excised and subjected to LC-MS/MS. A total of 776 cellular proteins were identified (Supplementary Table 1) and used for bioinformatics analysis. GO analysis showed that there were three annotations containing biological processes, cellular components, and molecular functions (Figure 1A). The biological processes were related to cellular component organization or biogenesis, chromatin assembly and nucleosome assembly, cellular component organization, and nucleosome organization (Figure 1A), while the cellular components were associated with organelle, nucleosome, DNA packing complex, and protein-DNA complex (Figure 1A). The molecular functions showed that binding was the most enriched, and structural molecular activity, structural constituent of ribosome, protein heterodimerization activity, and protein dimerization activity were also involved (Figure 1A). KEGG pathway enrichment analysis revealed an enrichment of 216 pathways. The top 20 prominent pathways, involved in systemic lupus erythematosus, necroptosis, vascular smooth muscle contraction, renin secretion, viral carcinogenesis, NOD-like receptor signaling, Gonadotropin-releasing hormone (GnRH) signaling, and inflammatory mediator regulation of transient receptor potential (TRP) channels were selected (Figures 1B–D). Interestingly, systemic lupus erythematosus, necroptosis, vascular smooth muscle contraction, and renin secretion pathways were found to be potentially related to PDNS induced by PCV3 infection.

### Mapping the Protein Interacting Network and Identifying the Cellular Proteins Interacting With PCV3 Cap

**FIGURE 2:**
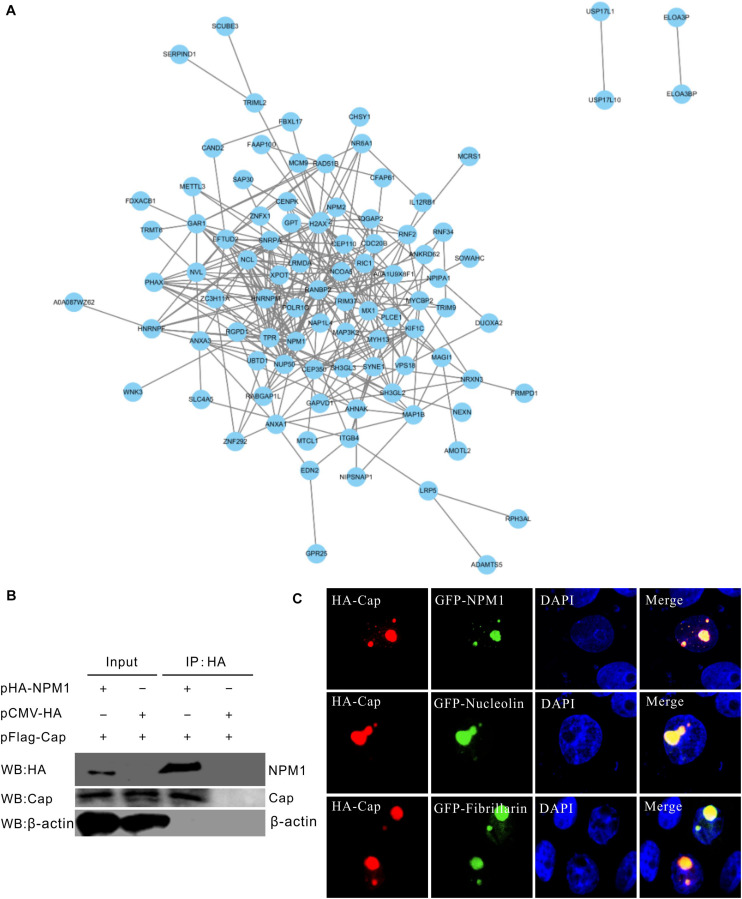
Mapping and identification of protein interactions. **(A)** Mapping of protein-protein interactions (PPIs) network using STRING database. Top ranking PPIs were selected to map the interaction network. The PPIs network with PCV3 Cap was constructed by Cytoscape software. **(B)** Confirmation of interaction between PCV3 Cap and NPM1. HEK293T cells were co-transfected with plasmids expressing FLAG-Cap and HA-NPM1. Co-transfection with an empty vector was used as a negative control. Cell lysates were prepared at 24 h after transfection and immunoprecipitated with HA beads. Protein samples were separated by SDS-PAGE and detected with antibodies against FLAG and Cap by western blotting. **(C)** Colocalization of PCV3 Cap with NPM1 and nucleolar proteins. PK15 cells were co-transfected with plasmids expressing HA-Cap with NPM1-GFP, nucleolin-GFP, and fibrillarin-GFP, respectively. The cells were incubated with the antibodies corresponding to the HA tag at 24 h post-transfection, and followed by the Alexa Fluor 568-conjugated secondary antibodies (red). Nuclei were stained with DAPI (blue).

STRING database was used to construct the PCV3 Cap protein and cellular protein interacting network. The protein-protein interactions (PPIs) showed that Cap interacts with several innate immune molecules, such as MX1, the family of zinc finger protein (ZNF), including ZNF292 and ZNF589, E3 ubiquitin ligase-like RING finger E3 ubiquitin ligase (TRIM9), and the E3 ligase RING finger protein 34 (RNF34) (Figure 2A). RNF34 is a negative regulator for the NOD1 pathway (Zhang et al., 2014). In addition, the components of nuclear pore complexes (NPCs), such as NUP50, NUP98, and nuclear receptor coactivator 5 (NCOA5), as well as the heterogeneous nuclear ribonucleoprotein (hnRNP), including hnRNP M and hnRNP F, and nucleolar protein nucleolin (NCL) and NPM1, all interacted with PCV3 Cap. Furthermore, NPM1 was found to interact with Cap by Co-IP in HEK293FT-cotransfected cells (Figure 2B). Moreover, in PK15 co-transfected cells, HA-Cap was found to perfectly colocalize with GFP-NPM1, GFP-nucleolin, and GFP-fibrillarin (Figure 2C). NPM1 has been demonstrated to interact with PCV2 Cap protein and contribute to PCV2 replication (Zhou et al., 2020). As reported in previous studies, in which PCV3 Cap was found to have a nucleolar localization signal (Mou et al., 2019), the present study also demonstrated that PCV3 Cap interacts and colocalizes with nucleolar phosphoprotein NPM1 (Zhou et al., 2021). Thus, we focused on the interaction between the PCV3 Cap protein and NPM1 and the contribution of NPM1 to PCV3 replication in subsequent experiments.

### The Region Responsible for the Interaction of Cap Protein With NPM1

**FIGURE 3:**
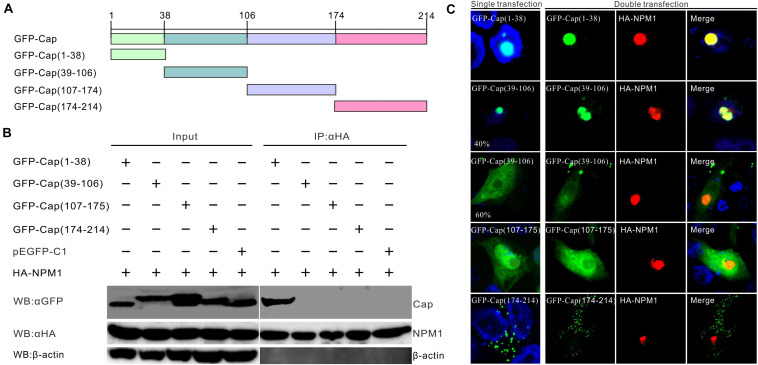
Determination of the binding domains of PCV3 Cap with NPM1. **(A)** Engineering of truncated constructs of PCV3 Cap, GFP-Cap (1–38), GFP-Cap (39–106), GFP-Cap (107–174), and GFP-Cap (175–214). **(B)** The interaction regions between NPM1 and Cap confirmed by Co-IP in HEK293FT cells with co-transfection. At 24 h post-transfection, the cell lysates were immunoprecipitated with Pierce Anti-HA Magnetic Beads, western blot analysis using antibodies to HA, GFP, and β-actin. **(C)** Colocalization analysis. PK15 cells were co-transfected with GFP-Cap, truncation constructs of GFP-Cap, and HA-NPM1. The cells were incubated with the antibodies corresponding to the HA tag at 24 h post-transfection, and followed by the Alexa Fluor 568-conjugated secondary antibodies (red). Nuclei were stained with DAPI (blue).

The Cap protein of PCV3 has a nucleolar localization signal at its N-terminal and is divided into four domains infused with GFP: GFP-Cap (1–38), GFP-Cap (39–106), GFP-Cap (107–174), and GFP-Cap (175–214) (Figure 3A). The truncated mutants expressed as GFP-fused proteins were co-transfected with HA-Cap in HEK293FT cells. The clarified cell supernatants were precipitated with Anti-HA Magnetic Beads. The results showed that GFP-Cap (1–38) was involved in the interaction, while aa.39–214 of Cap was not, demonstrating that the NLS domain of Cap interacted with NPM1 (Figure 3B). In transfected PK15 cells, the distribution of GFP-Cap (1–38) was the same as GFP-Cap in the nucleolus (Figure 3C). A part of GFP-Cap (39–106) was localized in the nucleolus, while others were found in the nucleus and cytoplasm. GFP-Cap (107–174) without NLS existed in the nucleus and cytoplasm, while GFP-Cap (175–214) was mainly found in the cytoplasm (Figure 3C). In co-transfected PK15 cells, GFP-Cap (1–38) was perfectly colocalized with HA-NPM1, while the other truncated GFP-Cap mutants did not colocalize with HA-NPM1 (Figure 3C). These results suggest that the N-terminus of PCV3 Cap interacts with NPM1.

### Cap Expression Resulted in the Redistribution of NPM1

**FIGURE 4:**
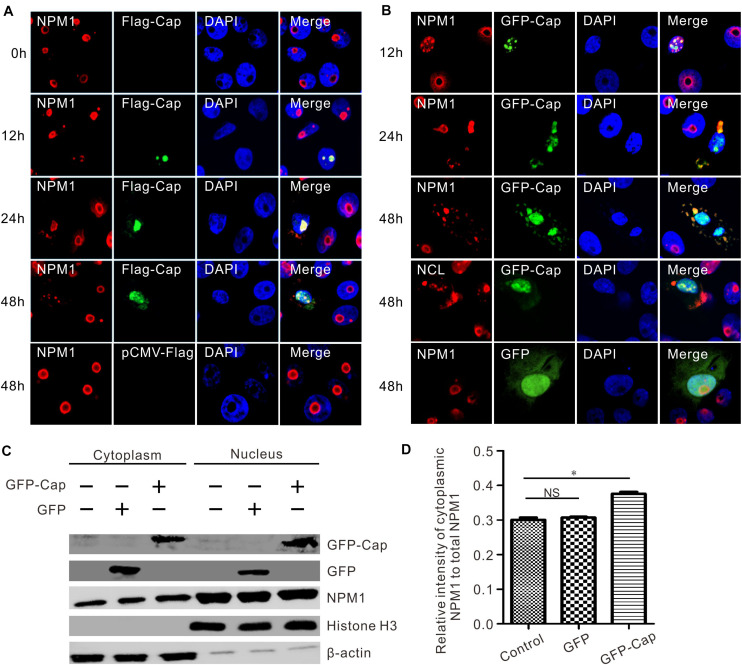
Endogenous colocalization analysis. PCV3 Cap induced the translocation of NPM1 to the cytoplasm from the nucleus. **(A,B)** PK15 cells were transfected to express FLAG-Cap and GFP-Cap. The cells were incubated with the antibodies corresponding FLAG tag, NPM1, and NCL followed by the Alexa Fluor 568-conjugated secondary antibodies (red). Nuclei were stained with DAPI (blue). **(C)** The cell nuclear and cytoplasmic extraction was performed after PK15 cells transfected GFP-Cap plasmid. At 48 h post-transfection, the protein samples were prepared and detected using antibodies against GFP and NPM1 by western blot analysis. Histone H3 and β-actin were used as fractionation quality controls. **(D)** The relative gray intensity of cytoplasmic NPM1 was calculated using ImageJ. Two-tailed Student’s *t*-test was used for statistical analysis. Each experiment was independently done three times. Data represent by mean ± standard deviation (SD). Asterisks (*) indicate the statistical significance: *P < 0.05; NS: not significant.

To observe the process of colocalization, PK15 cells were transfected with FLAG-Cap or GFP-Cap, and fixed at 12, 24, and 48 h post-transfection. The endogenous localization of NPM1 was detected by red immunofluorescence. The colocalization of FLAG-Cap or GFP-Cap and NPM1 was observed by confocal microscopy at 12 h post-transfection (Figures 4A,B), and both presented a typical nucleolar distribution at 12 h post-transfection (Figures 4A,B). However, the shape and localization of endogenous NPM1 changed at 24 h post-transfection. A part of NPM1 was translocated from the nucleolus into the cytoplasm and colocalized with FLAG-Cap or GFP-Cap. Moreover, the fluorescence of NPM1 was condensed in the middle of the nuclei, and the shape of NPM1 changed (Figures 4A,B). At 48 h post-transfection, the typical nucleolus distribution of FLAG-Cap or GFP-Cap and NPM1 in co-transfected cells disappeared, and they colocalized in the nucleus and cytoplasm. The distribution of nucleolin also changed in the GFP-Cap plasmid-transfected cells (Figure 4B). To evaluate the roles of GFP-Cap-induced translocation, a cytoplasmic-nuclear fractionation assay was used to measure their cellular distribution. To this end, PK15 cells were transfected with GFP-Cap and pEGFP-C1 vector, and the cytoplasmic and nuclear fractions were isolated 48 h post-transfection (Figure 4C). Cytoplasmic β-actin and nuclear histone subunit H3 were used as indicators of the quality of fractionation (Figure 4C). Compared with the control and pEGFP-C1 vector transfection, NPM1 was enriched in the cytoplasmic fraction after GFP-Cap transfection (Figure 4D). These results suggest that the transfection of FLAG-Cap or GFP-Cap did not affect the morphological integrity of the nucleolus in earlier stages, but changed the appearance and distribution of NPM1 in later stages.

### NPM1 Expression Is Upregulated Upon PCV3 Infection

**FIGURE 5:**
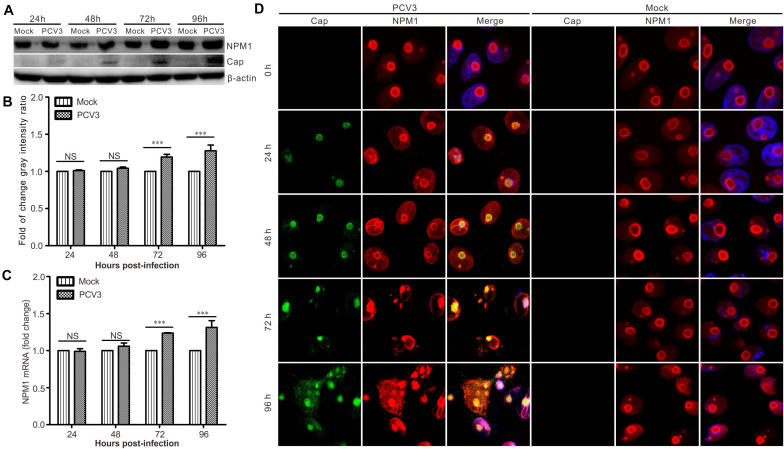
PCV3 increased the overall abundance of NPM1 in infected PK15 cells. **(A)** PK15 cells were infected with PCV3 and harvested at 24, 48, 72, and 96 h post-infection. The cell lysates were subjected to western blotting to examine the levels of Cap and NPM1 protein. β-actin was used as an internal control. **(B)** Graph showing the levels of NPM1 normalized against β-actin. The relative gray intensity was quantified using ImageJ. Each experiment was independently done three times. Data represent by the mean ± standard deviation (SD) (***P < 0.001; NS, not significant). **(C)** Real-time quantitative PCR was used to calculate the expression of NPM1 mRNA in PCV3-infected cells at 24, 48, 72, and 96 hpi. Expression was normalized to the *GAPDH* mRNA level. The relative expression was analyzed using GraphPad Prism. Each experiment was independently done three times. Data represent the mean ± SD (*P < 0.05; **P < 0.01; ***P < 0.01; NS, not significant). **(D)** PK15 cells were infected with PCV3, fixed, and subjected to immunofluorescence assay at 0, 24, 48, 72, and 96 hpi, respectively. Immunofluorescence signals revealed the expression of Cap (green) and NPM1 protein (red). DAPI was used to stain cell nuclei (blue).

Western blotting and RT-qPCR assays were used to determine the expression of NPM1 during PCV3 infection. The results showed that the levels of NPM1 protein expression and mRNA transcription increased significantly in PCV3-infected PK15 cells at 48, 72, and 96 h post-infection (Figures 5A–C). Confocal microscopy showed that the level of NPM1 protein expression increased in a time-dependent manner (Figure 5D). Initially, NPM1 was primarily localized to the nucleolus before PCV3 infection and was found to exhibit a ring distribution (Figure 5D). However, after PCV3 infection, the shape of the nucleolus changed, the fluorescence signals were enhanced, and part of NPM1 was translocated to the cytoplasm from the nucleus (Figure 5D). These results were consistent with those of transfection and suggest that PCV3 infection can enhance NPM1 expression, indicating the potential role of NPM1 in PCV3 infection.

### NPM1 Overexpression in PK15 Cells Promotes PCV3 Replication

**FIGURE 6:**
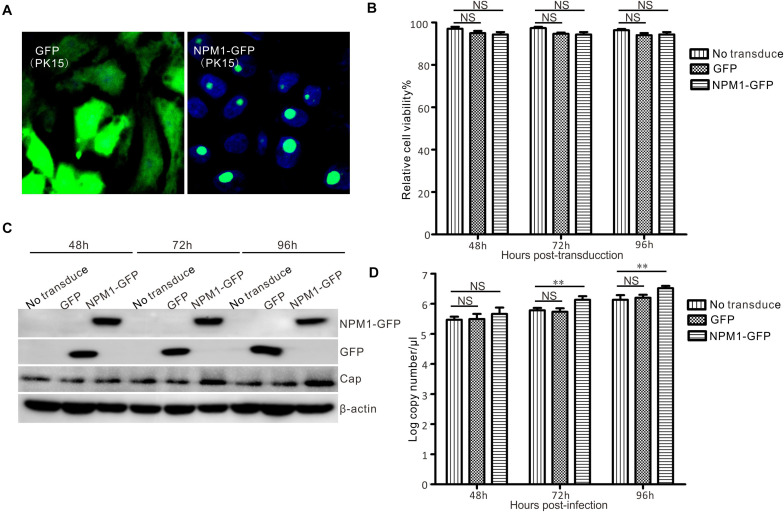
Enhancement of PCV3 replication by NPM1 overexpression in lentivirus-transduced PK15 cells. **(A)** Lentiviruses were packed in HEK293FT cells, and the lentiviruses of NPM1-GFP and GFP transduced into PK15 cells were examined by microscopy. **(B)** The viability of lentivirus-transduced PK15 cells was analyzed by CCK-8 assay at 48, 72, and 96 h post-transduction. **(C)** Lentivirus-transduced PK15 cells and no-transduced PK15 cells were infected with PCV3. The infected cells were harvested at 48, 72, and 96 h post-infection. Western blotting was used to examine the expression levels of Cap, NPM1-GFP, GFP, and β-actin. **(D)** The copy numbers of viral DNA were determined by qPCR. Data represent the mean ± standard deviation (SD) of three independent experiments (**P < 0.01; NS, not significant).

To determine the effect of NPM1 on the replication of PCV3, NPM1-GFP, or GFP PK15 cells stably expressing NPM1-GFP or GFP were established using a lentiviral system. Subsequently, immunofluorescence microscopy and western blotting showed that NPM1-GFP and GFP were expressed at similar levels (Figures 6A,C). Cell growth and viability were also similar (Figure 6B). Western blot analysis showed a higher intensity lane of Cap in NPM1-GFP expressing cells than in GFP-expressing cells and no-transduced cells at 72–96 h after PCV3 infection. The copy numbers of viral DNA in the NPM1 overexpressed cells were higher than those in the GFP overexpressing cells and no-transduced cells at 72–96 h post-infection (Figure 6D). The results demonstrate that the overexpression of NPM1 enhances PCV3 replication.

### siRNA Knockdown or NPM1 Inhibitor Treatment Inhibits PCV3 Replication

**FIGURE 7:**
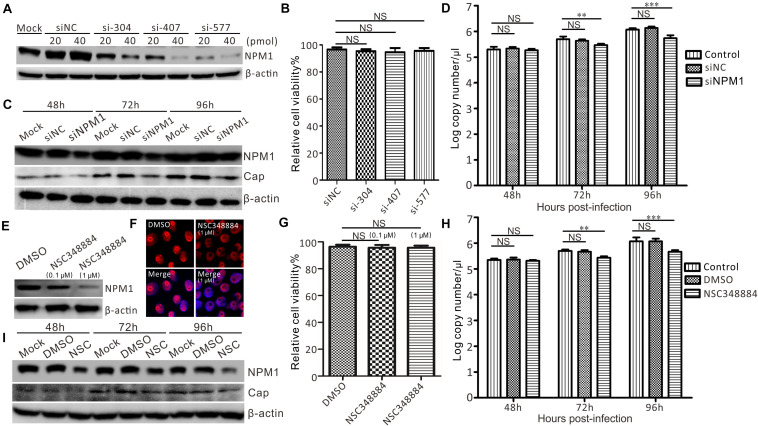
Suppression of PCV3 proliferation by silencing NPM1 expression in PK15 cells. **(A)** Silencing efficiency of NPM1 expression with different concentrations of specific siRNA (siNPM1). PK15 cells were transfected with siRNA for NPM1 at a concentration of 20 or 40 pmol. siNC and untransfected cells were used as negative controls. **(B)** Viability of NPM1 gene-silenced PK15 cells. **(C)** PCV3 replication in NPM1 gene-silenced PK15 cells by transfecting siNPM1-577. Cells were infected with PCV3 and harvested at 48, 72, and 96 h post-infection. Western blot was exploited to determine the expression levels of Cap, NPM1, and β-actin. **(D)** The copy numbers of viral DNA were determined by qPCR in siNPM1-577-transfected PK15 cells. Data represent the mean ± standard deviation (SD) of three independent experiments (**P < 0.01; ***P < 0.001; NS, not significant). **(E)** NPM1 inhibitor NSC348884 and DMSO-treated cells were infected with PCV3 for 48, 72, and 96 h. Western blot was used to examine the expression levels of Cap, NPM1, and β-actin. **(F)** NPM1 expression in NSC348884- and DMSO-treated cells analyzed by immunofluorescence. **(G)** Viability of PK15 cells treated with NSC348884 and DMSO analyzed using a CCK-8 assay. **(H)** qPCR was exploited to determine the expression level of Cap gene. Data represent the mean ± SD with three independent experiments (**P < 0.01; ***P < 0.001; NS, not significant). **(I)** PCV3 replication in NPM1 inhibitor-treated cells. DMSO-treated cells were used as a control. Western blotting was exploited to determine the expression levels of Cap, NPM1, and β-actin.

Given that the overexpression of NPM1 may promote PCV3 replication, the effect of NPM1 silencing on the proliferation of PCV3 was tested using siRNAs-mediated knockdown and NPM1 oligomerization inhibitors (NSC348884). Compared with the untransfected cells and the cells transfected with siNC, PK15 cells transfected with siNPM1 exhibited an efficient knockdown. A dose-dependent relationship was observed, and a final concentration of 20 pmol was used (Figure 7A). The viability of NPM1-silenced cells was similar to that of the control siRNA-transfected cells (Figure 7B). Western blot analysis showed that the expression of Cap was significantly reduced in siNPM1-577 transfected cells compared with that in untransfected cells and siNC control cells after PCV3 infection (Figure 7C). Furthermore, the virus copy numbers of Cap in the NPM1-silenced cells were lower than those in non-transfected PCV3-infected cells and siNC control cells after PCV3 infection (Figure 7D).

PK15 cells were treated with NSC348884 and harvested to examine the expression of NPM1. Compared with the DMSO-treated cells, PK15 cells treated with NSC348884 exhibited effective inhibition in a dose-dependent fashion (Figure 7E). The endogenous distribution of NPM1 was detected by red immunofluorescence. The typical morphological integrity of the nucleolus was affected in PK15 cells after treatment with NSC348884 (Figure 7F), while the viability of NSC348884-treated cells was similar to that of the DMSO-treated cells (Figure 7G). The results showed that the expression of Cap was reduced in NSC348884-treated cells compared with that in DMSO-treated cells and normal cells after PCV3 infection (Figure 7G), and the virus copy numbers of Cap in NSC348884-treated cells were lower than those in the normal and DMSO-treated cells after PCV3 infection (Figure 7H). Western blot analysis showed that the expression of Cap was significantly reduced in NSC348884-treated cells compared with that in DMSO-treated cells and normal cells after PCV3 infection (Figure 7I).

## Discussion

The nucleolus is the most remarkable component of the nucleus, acting as the site for rRNA biogenesis and ribosome assembly in eukaryotes. Metabolic disorders and viral infections have been shown to promote alterations of the nucleolus structure (Greco, 2009; Hiscox et al., 2010; Salvetti and Greco, 2014). In recent years, research has increasingly revealed that various types of viruses can hijack the nucleus, particularly the proteins of the nucleolus, for their own replication and pathogenic processes (Sirri et al., 1997; Huang et al., 2001; Hiscox et al., 2010). During viral infection, viral-encoded proteins containing nucleolar localization signal can shuttle to and from the nucleolus, where they interact with nucleolar cellular proteins, inducing the redistribution or modification of nucleolar proteins. By acting as a multifunctional nucleolar protein, NPM1 participates in many steps of the viral life cycle, including entry into the nucleus, viral genome transcription and replication, capsid assembly, and egress by binding to viral proteins with nucleolar localization signals (Liu et al., 2012; Jeong et al., 2014; Day et al., 2015; Nouri et al., 2015). With regards to PCV3, a recent study found that the interaction of NPM1 with PCV3 Cap contributes to its nucleolar localization (Zhou et al., 2021). However, whether the regulation of NPM1 is involved in PCV3 replication in this context remains to be elucidated. In this study, immunoprecipitation coupled with LC-MS/MS was used to identify the host cellular proteins interacting with PCV3 Cap. As a result, NPM1 was found to interact with Cap at its N-terminus. Moreover, infection with PCV3 was found to upregulate the expression of NPM1 in cultured PK15 cells, while the expression of Cap protein induced NPM1 redistribution from the nucleus to the cytoplasm. Silencing NPM1 expression resulted in a significant reduction in PCV3 replication, whereas NPM1 overexpression was found to promote PCV3 replication. These results suggest that NPM1 interacts with PCV3 Cap and plays a pivotal role in the replication of PCV3.

In this study, nucleolar protein NPM1 was identified as a PCV3 capsid-interacting factor by IP/LC-MS and Co-IP (Figures 1, 2). The NPM1-binding ability of Cap protein was mapped to a region occurred at amino acids 1–38 of the N-terminus (Figure 3), consistent with the results obtained in a recent report (Zhou et al., 2021). Confocal microscopy indicated that PCV3 Cap and NPM1 were both localized to the nucleus, in addition to the nucleolus, after PCV3 infection or co-transfection (Figures 3, 4). NPM1 is a multifunctional protein having ribonuclease, nucleic acid-binding, and molecular chaperone activities (Huang et al., 2001). Multiple studies have shown that the C-terminal of NPM1 interacts with viral proteins (Adachi et al., 1993; Li, 1997). For example, amino acids 194–239 of NPM1 containing the NLS-binding domain are required for its interaction with HIV Tat protein (Li, 1997). The N-terminal 19-amino acid region of HTLV-1Rex protein containing nucleolar targeting signal (NOS) was identified to bind to NPM1. In addition, amino acids 120–132 and 161–188 of NPM1 act as interacting regions for NOS (Adachi et al., 1993), while amino acids 118–188 of NPM1 are essential for interacting with porcine epidemic diarrhea virus nucleocapsid containing the NLS (Shi et al., 2017). Furthermore, residues 188–245 of NPM1 have been found to be indispensable for the interaction with the M protein of Newcastle disease virus (NDV) (Duan et al., 2014). In contrast, the region interacting with Japanese encephalitis virus (JEV) core protein was found to be located at amino acid residues 38–77 in the N-terminus of NPM1 (Tsuda et al., 2006). Similarly, the binding regions with PCV2 and PCV3 Caps were found to be located at the N-terminus of NPM1, and the amino acid residue serine-48 was found to be dispensable for PCV2 or PCV3 Cap/NPM1 interaction (Finsterbusch et al., 2009; Zhou et al., 2020, 2021). However, further study is required to determine the contribution of the interaction between Cap and NPM1 containing serine-48 in PCV3 replication.

NPM1 translocation from the nucleoli to the cytoplasm was observed in PK15 cells infected with PCV3 (Figure 5). The redistribution of NPM1 to other cellular compartments has been previously shown to be important for the life cycle of the virus (Hiscox, 2002). For instance, adeno-associated virus capsid protein was found to force the translocation of NPM1 to the cytoplasm in virus trafficking and transduction processes (Johnson and Samulski, 2009). HSV-1 UL24 protein induces NPM1 dispersal to the cytoplasm, contributing to the nuclear egress of virions (Lymberopoulos et al., 2011). Similarly, adenoviral protein V was found to promote NPM1 translocation to the nucleoplasm, which is related to adenoviral replication (Ugai et al., 2012). Additionally, Schmallenberg virus (SBV) non-structural protein NSs were found to co-localize with NPM1 and fibrillarin, whereas SBV infection induced the relocalization of NPM1 from the nucleolus to the nucleoplasm (Gouzil et al., 2017). A fraction of NPM1 in the nucleoli has also been previously found to translocate to the cytoplasm during JEV infection (Tsuda et al., 2006). In the present study, the precise mechanism underlying NPM1 retention in the cytoplasm after PCV3 infection was not determined. However, based on our findings, we propose a hypothesis. A recent study revealed that infection with PCV3, Cap protein expressed in the cytoplasm and, to some degree, in the nucleus (Jiang et al., 2019). Thus, the cytoplasmic factors induced by PCV3 infection may capture NPM1, forcing its distribution in the cytoplasm of PCV3-infected cells. Thus, it will be interesting to determine which cytoplasmic factors induced by PCV3 infection are involved in the retention of NPM1 in the cytoplasm.

The contribution of NPM1 to viral replication has been demonstrated in numerous viruses, including hepatitis delta virus (Huang et al., 2001), adenovirus (Okuwaki et al., 2001), JEV (Tsuda et al., 2006), NDV (Duan et al., 2014), nervous necrosis virus (Mai et al., 2017), and PCV2 (Zhou et al., 2020). Although different viruses may cause varying redistributions of NPM1 in cellular locations, the depletion or overexpression of NPM1 is known to affect viral replication (Duan et al., 2014; Jeong et al., 2014). In the present study, the involvement of NPM1 in PCV3 replication was also confirmed by the fact that the siRNA-mediated depletion or NPM1 inhibitor treatment markedly reduced the levels of PCV3 viral DNA synthesis and protein expression, while NPM1 overexpression significantly increased viral DNA levels and protein expression (Figures 6, 7). These findings demonstrate the important role of NPM1 in PCV3 replication. Thus, we hypothesized that regulation of PCV3 replication may be induced by altering the structure and function of NPM1, including alterations in host transcription, ribosome biogenesis, and protein synthesis (Colombo et al., 2011; Lindstrom, 2011). In the PPI network, the interaction of NUP50 and NUP98 with Cap indicated that entry of PCV3 Cap into the nucleus may allow for its interaction with the NPCs (Figure 2), while hnRNP M and hnRNP F in the nucleus may contribute to the transportation of PCV3 Cap and the nucleolar proteins NPM1 and NCL in the nucleolus, facilitating PCV3 replication (Figure 2). However, further studies on the alterations in proteomics and host transcription will be needed to determine the precise role of NPM1 in PCV3 replication.

In summary, the N-terminal amino acids 1–38 of PCV3 Cap were found to bind directly to NPM1, translocating to the cytoplasm from the nucleolus in PCV3-infected cells, demonstrating the contribution of NPM1 to PCV3 replication. Taken together, the results presented in this study provide important information for further understanding the mechanism of PCV3 replication. Thus, this work facilitates the utilization of NPM1 as a potential target for therapy and for the prevention of PCV3 infection.

## Data Availability Statement

The datasets presented in this study can be found in online repositories. The names of the repository/repositories and accession number(s) can be found in the article/Supplementary Material.

## Author Contributions

JL and JS conceived, designed the experiments, and drafted the manuscript revised the manuscript. JS, LW, HJ, JW, RS, and DW performed the experiments. RQ, SZ, JS, and LH were responsible for the statistical analysis of the data. All authors reviewed the manuscript.

## Conflict of Interest

The authors declare that the research was conducted in the absence of any commercial or financial relationships that could be construed as a potential conflict of interest.

## References

[B1] Adachi Y. Copeland T. D. Hatanaka M. Oroszlan S. (1993). Nucleolar targeting signal of Rex protein of human T-cell leukemia virus type I specifically binds to nucleolar shuttle protein B-23. *J. Biol. Chem.* 268 13930–13934. 10.1016/s0021-9258(19)85191-8 8314759

[B2] Breitbart M. Delwart E. Rosario K. Segales J. Varsani A. Ictv Report C. (2017). ICTV virus taxonomy profile: circoviridae. *J. Gen. Virol.* 98 1997–1998. 10.1099/jgv.0.000871 28786778 PMC5656780

[B3] Colombo E. Alcalay M. Pelicci P. G. (2011). Nucleophosmin and its complex network: a possible therapeutic target in hematological diseases. *Oncogene* 30 2595–2609. 10.1038/onc.2010.646 21278791

[B4] Day P. M. Thompson C. D. Pang Y. Y. Lowy D. R. Schiller J. T. (2015). Involvement of Nucleophosmin (NPM1/B23) in assembly of infectious HPV16 capsids. *Papillomavirus Res.* 1 74–89. 10.1016/j.pvr.2015.06.005 27398412 PMC4934132

[B5] Duan Z. Q. Chen J. Xu H. X. Zhu J. Li Q. H. He L. (2014). The nucleolar phosphoprotein B23 targets Newcastle disease virus matrix protein to the nucleoli and facilitates viral replication. *Virology* 452 212–222. 10.1016/j.virol.2014.01.011 24606698

[B6] Fankhauser C. Izaurralde E. Adachi Y. Wingfield P. Laemmli U. K. (1991). Specific complex of human immunodeficiency virus type 1 rev and nucleolar B23 proteins: dissociation by the Rev response element. *Mol. Cell Biol.* 11 2567–2575. 10.1128/mcb.11.5.2567 2017166 PMC360026

[B7] Finsterbusch T. Steinfeldt T. Doberstein K. Rodner C. Mankertz A. (2009). Interaction of the replication proteins and the capsid protein of porcine circovirus type 1 and 2 with host proteins. *Virology* 386 122–131. 10.1016/j.virol.2008.12.039 19178923

[B8] Gouzil J. Fablet A. Lara E. Caignard G. Cochet M. Kundlacz C. (2017). Nonstructural Protein NSs of schmallenberg virus is targeted to the nucleolus and induces nucleolar disorganization. *J. Virol.* 91 e1263–16. 10.1128/JVI.01263-16 PMC5165206 27795408

[B9] Greco A. (2009). Involvement of the nucleolus in replication of human viruses. *Rev. Med. Virol.* 19 201–214. 10.1002/rmv.614 19399920 PMC7169183

[B10] Heath L. Williamson A. L. Rybicki E. P. (2006). The capsid protein of beak and feather disease virus binds to the viral DNA and is responsible for transporting the replication-associated protein into the nucleus. *J. Virol.* 80 7219–7225. 10.1128/jvi.02559-05 16809327 PMC1489033

[B11] Hiscox J. A. (2002). The nucleolus–a gateway to viral infection? *Arch. Virol.* 147 1077–1089. 10.1007/s00705-001-0792-0 12111420 PMC7087241

[B12] Hiscox J. A. Whitehouse A. Matthews D. A. (2010). Nucleolar proteomics and viral infection. *Proteomics* 10 4077–4086. 10.1002/pmic.201000251 20661956 PMC7167898

[B13] Huang W. H. Yung B. Y. Syu W. J. Lee Y. H. (2001). The nucleolar phosphoprotein B23 interacts with hepatitis delta antigens and modulates the hepatitis delta virus RNA replication. *J. Biol. Chem.* 276 25166–25175. 10.1074/jbc.m010087200 11309377

[B14] Jeong H. Cho M. H. Park S. G. Jung G. (2014). Interaction between nucleophosmin and HBV core protein increases HBV capsid assembly. *Febs Lett.* 588 851–858. 10.1016/j.febslet.2014.01.020 24462683

[B15] Jiang H. Wang D. Wang J. Zhu S. She R. Ren X. (2019). Induction of porcine dermatitis and nephropathy syndrome in piglets by infection with porcine Circovirus Type 3. *J. Virol.* 93:e2045–18. 10.1128/JVI.02045-18 PMC6363995 30487279

[B16] Johnson J. S. Samulski R. J. (2009). Enhancement of adeno-associated virus infection by mobilizing capsids into and out of the nucleolus. *J. Virol.* 83 2632–2644. 10.1128/jvi.02309-08 19109385 PMC2648275

[B17] Li X. Bai Y. Zhang H. Zheng D. Wang T. Wang Y. (2018). Production of a monoclonal antibody against Porcine circovirus type 3 cap protein. *J. Virol. Methods* 261 10–13. 10.1016/j.jviromet.2018.07.014 30063907

[B18] Li Y. P. (1997). Protein B23 is an important human factor for the nucleolar localization of the human immunodeficiency virus protein Tat. *J. Virol.* 71 4098–4102. 10.1128/jvi.71.5.4098-4102.1997 9094689 PMC191564

[B19] Lindstrom M. S. (2011). NPM1/B23: a multifunctional chaperone in ribosome biogenesis and chromatin remodeling. *Biochem. Res. Int.* 2011:195209. 21152184 10.1155/2011/195209 PMC2989734

[B20] Liu C. D. Chen Y. L. Min Y. L. Zhao B. Cheng C. P. Kang M. S. (2012). The nuclear chaperone nucleophosmin escorts an epstein-barr virus nuclear antigen to establish transcriptional cascades for latent infection in human B cells. *PLoS Pathog.* 8:e1003084. 10.1371/journal.ppat.1003084 23271972 PMC3521654

[B21] Liu Q. Tikoo S. K. Babiuk L. A. (2001). Nuclear localization of the ORF2 protein encoded by porcine circovirus type 2. *Virology* 285 91–99. 10.1006/viro.2001.0922 11414809

[B22] Lymberopoulos M. H. Bourget A. Ben Abdeljelil N. Pearson A. (2011). Involvement of the UL24 protein in herpes simplex virus 1-induced dispersal of B23 and in nuclear egress. *Virology* 412 341–348. 10.1016/j.virol.2011.01.016 21316727

[B23] Mai W. J. Huang F. Chen H. Q. Zhou Y. J. Chen Y. (2017). Nervous necrosis virus capsid protein exploits nucleolar phosphoprotein Nucleophosmin (B23) function for viral replication. *Virus Res.* 230 1–6. 10.1016/j.virusres.2016.12.015 28034778

[B24] Matthews D. A. (2001). Adenovirus protein V induces redistribution of nucleolin and B23 from nucleolus to cytoplasm. *J. Virol.* 75 1031–1038. 10.1128/jvi.75.2.1031-1038.2001 11134316 PMC113999

[B25] Mou C. Wang M. Pan S. Chen Z. (2019). Identification of nuclear localization signals in the ORF2 protein of porcine circovirus type 3. *Viruses* 11:1086. 10.3390/v11121086 31766638 PMC6950156

[B26] Nouri K. Moll J. M. Milroy L. G. Hain A. Dvorsky R. Amin E. (2015). Biophysical characterization of nucleophosmin interactions with human immunodeficiency virus rev and herpes simplex virus US11. *PLoS One* 10:e0143634. 10.1371/journal.pone.0143634 26624888 PMC4704560

[B27] Oh T. Chae C. (2020). First isolation and genetic characterization of porcine circovirus type 3 using primary porcine kidney cells. *Vet. Microbiol.* 241:108576. 10.1016/j.vetmic.2020.108576 31928694

[B28] Okuwaki M. (2008). The structure and functions of NPM1/Nucleophsmin/B23, a multifunctional nucleolar acidic protein. *J. Biochem.* 143 441–448. 10.1093/jb/mvm222 18024471

[B29] Okuwaki M. Iwamatsu A. Tsujimoto M. Nagata K. (2001). Identification of nucleophosmin/B23, an acidic nucleolar protein, as a stimulatory factor for in vitro replication of adenovirus DNA complexed with viral basic core proteins. *J. Mol. Biol.* 311 41–55. 10.1006/jmbi.2001.4812 11469856

[B30] Palinski R. Pineyro P. Shang P. Yuan F. Guo R. Fang Y. (2017). A novel porcine circovirus distantly related to known circoviruses is associated with porcine dermatitis and nephropathy syndrome and reproductive failure. *J. Virol.* 91 e1879–16. 10.1128/JVI.01879-16 PMC5165205 27795441

[B31] Passos-Castilho A. M. Marchand C. Archambault D. (2018). B23/nucleophosmin interacts with bovine immunodeficiency virus Rev protein and facilitates viral replication. *Virology* 515 158–164. 10.1016/j.virol.2017.12.021 29289827

[B32] Phan T. G. Giannitti F. Rossow S. Marthaler D. Knutson T. P. Li L. (2016). Detection of a novel circovirus PCV3 in pigs with cardiac and multi-systemic inflammation. *Virol. J.* 13:184. 27835942 10.1186/s12985-016-0642-z PMC5105309

[B33] Ramirez-Boo M. Nunez E. Jorge I. Navarro P. Fernandes L. T. Segales J. (2011). Quantitative proteomics by 2-DE, 16O/18O labelling and linear ion trap mass spectrometry analysis of lymph nodes from piglets inoculated by porcine circovirus type 2. *Proteomics* 11 3452–3469. 10.1002/pmic.201000610 21751353

[B34] Salvetti A. Greco A. (2014). Viruses and the nucleolus: the fatal attraction. *Biochim. Biophys. Acta* 1842 840–847. 10.1016/j.bbadis.2013.12.010 24378568 PMC7135015

[B35] Samad M. A. Okuwaki M. Haruki H. Nagata K. (2007). Physical and functional interaction between a nucleolar protein nucleophosmin/B23 and adenovirus basic core proteins. *Febs Lett.* 581 3283–3288. 10.1016/j.febslet.2007.06.024 17602943

[B36] Schmittgen T. D. Livak K. J. (2008). Analyzing real-time PCR data by the comparative C-T method. *Nat. Protoc.* 3 1101–1108. 10.1038/nprot.2008.73 18546601

[B37] Shi D. Shi H. Y. Sun D. B. Chen J. F. Zhang X. Wang X. B. (2017). Nucleocapsid Interacts with NPM1 and protects it from proteolytic cleavage, enhancing cell survival, and is involved in PEDV growth. *Sci. Rep.* 7:39700. 28045037 10.1038/srep39700 PMC5206633

[B38] Shuai J. Wei W. Jiang L. Li X. Chen N. Fang W. (2008). Mapping of the nuclear localization signals in open reading frame 2 protein from porcine circovirus type 1. *Acta Biochim. Biophys. Sin (Shanghai)* 40 71–77. 10.1111/j.1745-7270.2008.00377.x 18180855

[B39] Sirri V. Roussel P. Gendron M. C. Hernandez-Verdun D. (1997). Amount of the two major Ag-NOR proteins, nucleolin, and protein B23 is cell-cycle dependent. *Cytometry* 28 147–156. 10.1002/(sici)1097-0320(19970601)28:2<147::aid-cyto8>3.0.co;2-c 9181305

[B40] Tsuda Y. Mori Y. Abe T. Yamashita T. Okamoto T. Ichimura T. (2006). Nucleolar protein B23 interacts with Japanese encephalitis virus core protein and participates in viral replication. *Microbiol. Immunol.* 50 225–234. 10.1111/j.1348-0421.2006.tb03789.x 16547420

[B41] Ugai H. Dobbins G. C. Wang M. Le L. P. Matthews D. A. Curiel D. T. (2012). Adenoviral protein V promotes a process of viral assembly through nucleophosmin 1. *Virology* 432 283–295. 10.1016/j.virol.2012.05.028 22717133 PMC3423539

[B42] Wurm T. Chen H. Hodgson T. Britton P. Brooks G. Hiscox J. A. (2001). Localization to the nucleolus is a common feature of coronavirus nucleoproteins, and the protein may disrupt host cell division. *J. Virol.* 75 9345–9356. 10.1128/jvi.75.19.9345-9356.2001 11533198 PMC114503

[B43] Yun J. P. Chew E. C. Liew C. T. Chan J. Y. H. Jin M. L. Ding M. X. (2003). Nucleophosmin/B23 is a proliferate shuttle protein associated with nuclear matrix. *J. Cell Biochem.* 90 1140–1148. 10.1002/jcb.10706 14635188

[B44] Zhang H. Guo X. Ge X. Chen Y. Sun Q. Yang H. (2009). Changes in the cellular proteins of pulmonary alveolar macrophage infected with porcine reproductive and respiratory syndrome virus by proteomics analysis. *J. Proteome Res.* 8 3091–3097. 10.1021/pr900002f 19341299

[B45] Zhang H. H. Hu W. Q. Li J. Y. Liu T. N. Zhou J. Y. Opriessnig T. (2020). Novel circovirus species identified in farmed pigs designated as Porcine circovirus 4, Hunan province, China. *Transbound Emerg. Dis.* 67 1057–1061. 10.1111/tbed.13446 31823481

[B46] Zhang R. Zhao J. Song Y. H. Wang X. Wang L. L. Xu J. (2014). The E3 Ligase RNF34 is a novel negative regulator of the NOD1 pathway. *Cell Physiol. Biochem.* 33 1954–1962. 10.1159/000362972 25012219

[B47] Zhou J. W. Dai Y. D. Lin C. Zhang Y. Feng Z. X. Dong W. R. (2020). Nucleolar protein NPM1 is essential for circovirus replication by binding to viral capsid. *Virulence* 11 1379–1393. 10.1080/21505594.2020.1832366 33073687 PMC7575006

[B48] Zhou J. W. Li J. Li H. M. Zhang Y. Dong W. R. Jin Y. L. (2021). The serine-48 residue of nucleolar phosphoprotein nucleophosmin-1 plays critical role in subcellular localization and interaction with porcine circovirus type 3 capsid protein. *Vet. Res.* 52:4. 33413620 10.1186/s13567-020-00876-9 PMC7792357

